# The Therapeutic Potential of the Restoration of the p53 Protein Family Members in the *EGFR*-Mutated Lung Cancer

**DOI:** 10.3390/ijms23137213

**Published:** 2022-06-29

**Authors:** Matilde Fregni, Yari Ciribilli, Joanna E. Zawacka-Pankau

**Affiliations:** 1 Department of Cellular, Computational, and Integrative Biology (CIBIO), University of Trento, 38123 Povo, TN, Italy; matilde.fregni@studenti.unitn.it (M.F.); yari.ciribilli@unitn.it (Y.C.); 2 Center for Hematology and Regenerative Medicine, Department of Medicine, Huddinge, Karolinska Institutet, 141 86 Stockholm, Sweden; 3 Chair and Department of Biochemistry, Medical University of Warsaw, 02-097 Warsaw, Poland

**Keywords:** lung cancer, *EGFR*, TKI resistance, molecular targeted therapies, p53, p73, drug repurposing

## Abstract

Despite the recent development of precision medicine and targeted therapies, lung cancer remains the top cause of cancer-related mortality worldwide. The patients diagnosed with metastatic disease have a five-year survival rate lower than 6%. In metastatic disease, *EGFR* is the most common driver of mutation, with the most common co-driver hitting *TP*53. *EGFR*-positive patients are offered the frontline treatment with tyrosine kinase inhibitors, yet the development of resistance and the lack of alternative therapies make this group of patients only fit for clinical trial participation. Since mutant p53 is the most common co-driver in the metastatic setting, therapies reactivating the p53 pathway might serve as a promising alternative therapeutic approach in patients who have developed a resistance to tyrosine kinase inhibitors. This review focuses on the molecular background of *EGFR*-mutated lung cancer and discusses novel therapeutic options converging on the reactivation of p53 tumor suppressor pathways.

## 1. Introduction

In 2020, the estimated number of new cancer cases was 19.3 million, followed by around 10 million cancer deaths. In the same year, lung cancer was the second most common cancer type (2.2 million new cases), at the same time representing the primary cause of cancer-related deaths (1.8 million) [1]. Data collected from GLOBACAN 2020 estimate that by 2050, lung cancer-related deaths will increase to 4 million [2]. In the United States and Europe, lung cancer incidence and deaths in men remain the top cause of cancer-related mortality, and, in women, lung cancer is the third most frequent cancer type after breast and colorectal cancer [1]. Due to its high mortality rate, lung cancer represents a significant burden. Indeed, patients with metastatic lung cancer (57% of all diagnosed cases) have a five-year survival of only around 6%. Recent approvals of new molecular targeted therapies and immunotherapies, particularly for non-small cell lung cancer (NSCLC), have improved the outcomes in patients with the localized lung stage I-II tumors; additionally five-year survival rates increased to 59% [3].

## 2. Risk Factors

An epidemiological study by Doll and Hill revealed the relationship between tobacco consumption and lung cancer. Eighty-seven percent of lung cancer deaths are attributed to smoking. Additionally, in the United States, according to The National Research Council, environmental tobacco smoke may be responsible for 2–3% of all lung cancer deaths [4]. Yet, the probability of developing lung cancer is halved in individuals who successfully quit smoking for 10–15 years compared to those who keep smoking [5]. Diverse additional environmental factors are believed to be responsible for the development of lung cancer, including biomass fuel, diesel exhaust, radon, and asbestos which confer a higher risk in smokers due to their synergistic effect with carcinogens found in cigarettes.

## 3. Histological Subtypes of Lung Cancer

In the past, four main histopathological subtypes have been described by the World Health Organization (WHO) classification system of lung cancer because of bronchial epithelium transformation: squamous cell carcinoma, adenocarcinoma, large cell (or undifferentiated) carcinoma, and small cell carcinoma (SCC). Due to the overlapping clinical features, squamous cell carcinoma, adenocarcinoma, and large cell carcinoma are collected and classified as non-small cell lung cancer (NSCLC) and comprise 40%, 25%, and 30%, respectively, of NSCLC cases [6,7,8]. The NSCLC represents around 80% of all lung cancer cases, whereas SCLC accounts for 15% of cases. The remaining 5% is represented by less common subtypes such as carcinoid tumors and carcinomas with pleomorphic, sarcomatoid, or sarcomatous elements [4]. According to the 2021 WHO classification of thoracic tumors, the list of lung tumors is much extended, yet it is still based on applying the morphology features first, and next supported by the immunohistochemistry and then the molecular profiling. Furthermore, in comparison with the 2015 WHO classification, more emphasis is placed upon the genetic profiling or recognition of the spread through airspaces (STAS) as a histologic feature with prognostic significance [9]. Since it is outside the scope of the current work to discuss all subtypes of lung cancer, we will focus on the most common subtype, which is non-small cell lung cancer.

## 4. Non-Small Cell Lung Cancer (NSCLC)

Different sub-types of cells can give rise to NSCLC. In example, squamous cell carcinoma develops from cells located in the internal part of the lungs and mainly from Type-I pneumocytes which cover around 95% of the internal surface of each alveolus. On the other hand, the cells in the alveoli that possess a secretory activity, the so-called Type-II pneumocytes, can give rise to adenocarcinoma. Instead, as the name suggests, large cell carcinoma can arise from different types of large cells which appear larger under the microscopic examination.

NSCLC is diagnosed through several methods, including chest X-ray, CT-scan, MRI scan and positron emission tomography (PET). For predictive biomarker assessment histopathology is performed using lung biopsy. For accurate biomarker evaluation for the precision medicine in lung cancer, the analysis of lung tissue by digital pathology and machine learning has recently been established. This approach is called artificial intelligence (AI) and allows for the automated analysis of the histopathological data in diagnostic pathology [10,11].

Since tissue biopsy is an invasive method linked to worse patients’ well-being, non-invasive methods for lung cancer diagnosis, prediction, and treatment monitoring have been established. Currently, apart from tissue biopsies, liquid biopsies are collected for genetic testing allowing for identification of actionable variants using next-generation sequencing (NGS). For this purpose, circulating tumor cells, cell-free DNA, and, most recently, extracellular vesicles are collected and analyzed. The suitability of liquid biopsies for biomarker identification has been broadly tested and, in June 2016, cobas EGFR Mutation Test v2 for the detection of exon 19 deletions or exon 21 missense mutations in the *EGFR* gene was approved by the FDA [12].

The staging of lung cancer at diagnosis is as follow: occult (hidden) stage, Stage 0 (represented by abnormal cells; it may be adenocarcinoma in situ (AIS) or squamous cell carcinoma in situ (SCIS)), Stage I (the cancer is located in the lungs and has not spread to the sentinel lymph nodes), Stage II (the cancer is bigger and may have spread to the sentinel lymph nodes), Stage III (the tumor mass is larger and some cancer cells have spread to the nearby tissues/organs), or Stage IV (cancer has metastasized to distant sites). The staging plays a pivotal role in the design of the therapy: the early stage lung cancer is treated with surgery and radiation, yet if the tumor is in an advanced stage, chemotherapy, immune therapy, and targeted therapy are applied as neoadjuvant treatment, in order to facilitate the surgery, or as an adjuvant treatment, after the surgery [13]. Nowadays, thanks to the development of precision medicine, the treatment decision is taken based on the predictive biomarkers for clear-cut patient stratification, such as *EGFR* (Epidermal Growth Factor Receptor) mutation status.

## 5. Driver Mutations in NSCLC

The first trials with tyrosine kinase inhibitors targeting *EGFR* in NSCLC were initiated in 2001, and, since that time, targeted therapies have emerged as an effective management strategy in lung cancer patients [14,15]. As a result, in 2004, screening for somatic *EGFR* mutations was introduced for the advanced NSCLC to stratify patients for the targeted therapy with tyrosine kinase inhibitors [16]. Due to the development of precision medicine approaches, key somatic mutations were identified in lung adenocarcinomas (LUADs) and lung squamous cell carcinomas (LUSCs) which are now classified as driver mutations [17]. These mutations include aberrations in *EGFR, KRAS, RB,* or *ALK* genes. In addition to driver mutations, the whole-exome sequencing and broadly targeted sequencing panels showed the presence of several co-occurring or mutually exclusive driver mutations [18,19,20,21,22,23,24]. The co-occurrence of driver mutations is associated with improved fitness of cancer cells, while the mutual exclusivity can be explained by redundancy or antagonism [25,26]. NSCLC subgroups are divided according to the driver mutations and are applied both as predictive biomarkers for treatment stratification and in the studies on lung cancer pathogenesis [27].

One of the most common oncogenic activation mutations occurs in the *KRAS* gene. KRAS is a signal transducer protein regulating the RAS/MAPK kinase pathway. Activating *KRAS* mutations are present in 25–32% of both early-stage and metastatic LUADs [19,21,24,28], which are biologically and clinically heterogeneous. Another most frequent driver mutation occurs within the tyrosine receptor EGFR and is mutually exclusive with KRAS oncogene activation [28]. In the majority of LUADs, *EGFR* mutations are accompanied by one or more co-mutations where the most representative are the ones targeting the *TP*53 encoding gene [18,21,29,30]. With *EGFR* being the most relevant in the context of metastatic disease, the review will focus on the description of clinically pertinent mutations occurring within this tyrosine kinase receptor and the current and novel therapeutic approaches developed to date.

## 6. Epidermal Growth Factor Receptor Mutations

**Figure 1:**
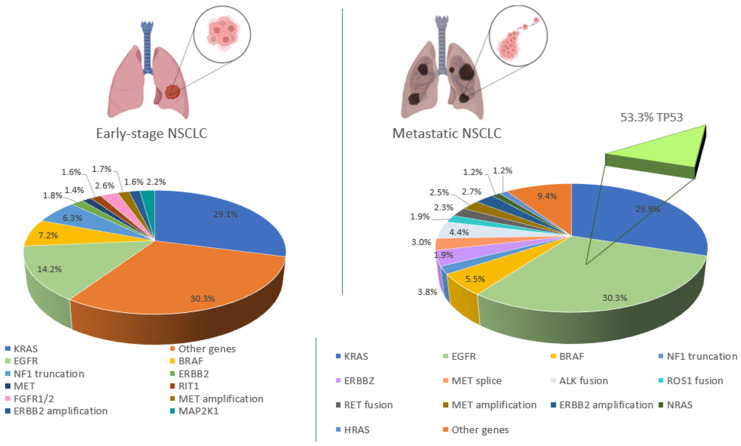
Prevalence of the driver mutations in localized and metastatic non-small cell lung adenocarcinoma. In the localized, early-stage non-small cell lung adenocarcinoma (**left** panel) the most prevalent mutations occur within *KARS* and *EGFR* genes. Other relevant genes, are *BRAF*, often mutated with *TP*53, *LKB*1, *ATM*, *NF*1, *PIK*3CA, *KEAP*1, *MYC* and *NKX*2-1 and *ERBB*2, often mutated with *NKX*2-1 amplification, *ERBB2* itself and in *RB1* mutations. Other relevant mutations of prevalence less then 1% are: (*HRAS*, *NRAS*, *RET* fusion, *ROS*1 fusion and *ALK* fusion for early-stage NSCLC and *RIT*1, *FGFR*1 or *FGFR*2 and *MAP*2K1 mutations for metastatic NSCLC). Some mutations, which have a low prevalence in the early stages, become more significant in metastatic settings (**right** panel), these mutations include *EGFR*, *ALK*, *ROS*1 and *RET* fusions and *MET* splice mutations. *ALK* rearrangements, as well as *ROS*1 and *RET* fusions, often co-occur with *CDKN*2A (32.5%) and *CDKN*2B (26.5%) mutations. *MET* exon 14 skipping is usually co-mutated with *MDM*2 and *CDK*4 amplification. In *EGFR*-mutated sub-group, the most common co-driver mutation is in *TP*53 gene (53.3%). Only mutations with a prevalence higher than 1% are shown in the picture. Data from early stage LUAD come from the combination of whole genome sequencing and data deriving from PanCancer Atlas cohort of The Cancer Genome Atlas (TCGA; *n* = 566) [31,32,33] and from the study of Imielinski and colleagues [25] and Kadara and co-workers [34], after the exclusion of stage IV patients. Data regarding the incidence of *MET* splice site alterations, *MET* amplification, *ERBB2* amplification and *ALK*, *ROS*1, and *RET* fusions derive only from TCGA and the study by Imielinski and collaborators. Advanced or metastatic LUAD driver mutation prevalence derives from next-generation sequencing of predefined panels from the Memorial Sloan Kettering Cancer Center [21] and from samples referred to the Foundation Medicine [18]. Data regarding alterations in *NF*1, *NRAS*, *HRAS*, *MAP2K*1, *FGFR*1, *FGFR*2 and *RIT*1 are based on MSK-IMPACT trial only.

In the localized, early-stage LUAD, the most prevalent mutations are *KRAS* mutations (29.1%) followed by *EGFR* (14.2%) mutations, as shown in Figure 1. Yet, the incidence of *EGFR* mutations doubles in metastatic disease (to around 30.3%), while the frequency of *KRAS* mutations remains constant (around 29%). Compared to early-stage disease, metastatic NSCLCs are significantly more heterogeneous, and additional driver mutations, of which the frequency is higher than 1%, have been detected in around 10% of patients. These additional drivers include *ALK*, *ROS*1, *RET* fusions, and *MET* splice mutations, which were shown to be negatively correlated with *KRAS* mutations. Yet, the range of enriched genomic co-alterations in advanced *EGFR*-mutant LUAD in majority includes recurrent mutations in *TP*53 (54.6–64.6%), *RB*1 (9.6–10.33%), *CTNNB*1 (5.3–9.6%), and *PIK*3CA (9–12.4%) [30].

## 7. Targeting Mutated EGFR

The Epidermal Growth Factor Receptor (EGFR, EC:2.7.10.1), also known as ErbB1/HER1, is a receptor tyrosine-protein kinase erbB-1 and a member of the tyrosine kinase receptor family. It was discovered in 1959, by the Nobel Prize winner Stanley Cohen, during the studies on the nerve growth factor using the whole extract of murine salivary glands.

Upon the binding of the ligand, epidermal growth factor (EGF), EGFR, activates two key pathways, PI3K/AKT/mTOR and RAS/RAF/MEK, involved in cell proliferation, cell survival, cell differentiation, as well as oncogene activation, and inhibition of apoptosis [35]. EGFR functions both as a signal-transducing receptor protein and as a transcription factor.

The gene encoding for EGFR is located at the short arm of chromosome 7, q22, spanning the 110 kb DNA region divided into 28 exons. EGFR is expressed in normal cells in up to 100,000 molecules, yet in specific cancer types, this number is elevated and can reach 10^6^ molecules per cell [36].

The protein is synthesized as a precursor, which is next cleaved at the N-terminus to form an 1186-residue-long mature form. EGFR is a complex protein, and it consists of an extracellular ligand binding and dimerization arm (exons 1–16), a hydrophobic transmembrane domain (exon 17), and the intracellular tyrosine kinase and C-terminal tail domains (exons 18–28) [37].

The tyrosine kinase domain, responsible for binding ATP, is also a site of the most common cancer-related mutations. *EGFR* gene is subject to activating mutation in NSCLC. Deletions in exon 19 (Ex19Del) and L858R mutation in exon 21 represent the so-called “classical *EGFR* mutations”, accounting for 85% of all mutations occurring in the receptor [38]. The remaining 10–20% mutations, also referred to as “rare *EGFR* mutations”, occur within or outside the kinase domain and consist of point mutations, deletions, and insertions within exons 18–25 [39]. An additional class of mutations is the so-called compound mutations, also known as complex, double or multiple mutations. As the name implies, more than one mutation occurs within the *EGFR* coding gene, either common or uncommon [40].

Structural studies demonstrated that the classical mutations destabilize the inactive conformation of EGFR monomer, promoting and increasing receptor dimerization and thus, activation. Leucine 858 is located in the activation loop within the helical turn and in wild-type EGFR it stays buried. The amino acid substitution L858R allows for R858 flipping out and the consequent interaction with the negatively charged residue E758 located in the intrinsically disordered αC-helix in the *N*-lobe in the monomeric EGFR. This triggers a shift in the αC-helix moving towards the ATP-ligand binding cleft, stabilizing in the so-called αC-in conformation category and thus the active state of the KD. Such motions promote the compaction and stabilization of the ATP binding site, as seen by a reduction in the distance between the αC-helix and the hinge region [41].

In Ex19Del EGFR, the amino acids, ^746^ELREA^750^, connecting the αC-helix with the β3 strand are deleted. This region is a flexible linker between αC and β3 and regulates the movements of αC-helix between αC-in and out conformations. The deletion of the segment impinges the β3-αC loop by shortening it, consequently preventing the rotation of the αC-helix [42,43,44] and stabilizing the active form, by reducing the flexibility of the αC-helix stabilized in the αC-in conformation.

Consequently, the compaction of the ATP binding site enables tighter binding of the tyrosine kinase inhibitors when compared to wild-type EGFR which is harnessed for the therapy of EGFR mutated NSCLC.

## 8. Tyrosine Kinase Inhibitors

The signal transduction by EGFR is complex and depends on ATP binding and transphosphorylation. Classical activating mutations such as exon 19 deletions and exon 21 L858R are associated with a lower affinity of EGFR for ATP compared to wild-type EGFR; therefore, first-generation EGFR inhibitors (EGFRi, gefitinib and erlotinib) have higher affinity to mutant EGFR when compared to wild-type protein and impede the receptor activation by effectively competing for the binding to the ATP binding pocket [42,45]. The gatekeeper mutation T790M has been clarified as a mechanism of resistance occurring in around 60% of the patients who did not respond to EGFRi [46]. This substitution increases the affinity for ATP, hampering the binding of TKIs [47].

To overcome the emergence of the resistance mutation, second-generation EGFRi (afatinib or dacomitinib, among others) was developed, which, unlike the first generation EGFRi which bind reversibly, bind covalently to the receptor, forming an irreversible complex surmounting the resistance arising from T790M. Unfortunately, second-generation EGFRi showed poor selectivity for L858R/T790M or EGFR/T790M mutant EGFR, failing in clinical trials [48,49,50,51]. On the other hand, third-generation EGFRi showed an increased selectivity for EGFR T790M when compared to second-generation EGFRi [52], leading to the rapid success of osimertinib in clinical trials. Osimertinib showed a superior median progression-free survival (PFS) of 17.2 months compared to 8.5 months for gefitinib or erlotinib in naïve NSCLC patients, leading to its approval by the FDA as a first-line treatment for patients with classical EGFR mutations in 2018 based on Phase III FLAURA clinical trial [53]. FLAURA is a double-blinded, randomized clinical trial of TAGRISSO^®^ (osimertinib) in 556 patients with locally advanced or metastatic, untreated NSCLC characterized by *EGFR* mutations [54]. The study showed that osimertinib increases the overall survival in *EGFR*-mutated advanced NSCLC patients [38,55].

The resistance toward EGFRi quickly arises and can be classified as either on-target or off-target. The most common mutation in *EGFR* associated with resistance is EGFR^C797S^. 14% on-target resistance mechanism emerges in patients that retained the T790M mutation after progression on osimertinib. Other, less common, on-target mutations are at positions G796, L792, L718, and G719 or G724, exon 20 insertions or *EGFR* gene amplifications [56].

Off-target resistance mechanisms are linked to *KRAS* mutations, *MET* amplification, *HER*2 amplification, *PIK*3CA mutation/amplification, and *PTEN* deletion. The therapeutic options for patients that relapsed on osimertinib are limited to combinations of chemotherapy with immunotherapy delivering variable outcomes.

Therefore, novel therapeutic approaches are needed for *EGFR*-mutated NSCLC that relapsed on TKIs. Promising, druggable targets in the group of patients that progressed on osimertinib are represented by mutant p53 and p73 tumor suppressor proteins.

## 9. p53 Tumor Suppressor

p53, also known as the guardian of the genome, plays a pivotal role in sensing the cellular stress and acts as a tumor suppressor through induction of the DNA repair or activation of genes involved in the cell cycle arrest, apoptosis, senescence, autophagy, ferroptosis or metabolism [57,58,59,60]. The p53 protein family, which includes p53 itself, p63, and p73, comprises domain structures and exists in different isoforms. The most common isoforms are the products of the transcription from two alternative promoters; P1 and P2. One of these isoforms, referred to as transcriptionally active (TA), acts as a tumor suppressor; in contrast, the other, referred to as N-terminus truncated isoform (ΔN), acts as an oncogene, for example, by acting as dominant-negative towards TA isoforms. The ratio between TA and ΔN isoforms is influenced by the degree of methylation of P1 and P2 promoters, and, in some cancer types, the TA/ΔN ratio can affect the efficacy of chemotherapy and radiotherapy (reviewed in [61]).

The gene encoding for p53 protein is composed of 13 exons, and it is located at the 17p13.1. The TA isoform is synthesized by the translation of 11 exons and consists of N-terminal, central, and C-terminal domains. The central DNA binding domain (DBD) allows p53 to recognize and bind to specific DNA sequences. The DBD has been highly conserved through evolution, and it is composed of an immunoglobulin-like β-sandwich scaffold, a loop-sheet-helix, and two large loops. The DBD is connected to the oligomerization domain, essential for p53 tetramerization. Finally, both the N-terminal, and C-terminal domains are unstructured and subject to numerous post-translational modifications. The N-terminal domain is a transactivation domain and the C-terminus is implied in DNA binding as well as in the recruitment of co-factors and regulation of p53 localization.

MDM2 is a major p53 E3 ubiquitin ligase, activated by either extrinsic or intrinsic signals, promoting p53 ubiquitination in the cytosol and the nucleus [62]. MDM2-mediated ubiquitination activates proteasomal degradation of p53, therefore, contributes to maintaining low levels of p53 in the absence of stress stimuli [62]. MDMX (also known as MDM4), instead, is a MDM2 homolog that lacks the E3 ligase activity towards p53 yet forms heterodimers with MDM2 and enhances its E3 ligase activity. Additionally, by binding to the N-terminal domain, MDM4 inhibits p53 transcriptional function [63].

Interestingly, MDM2 is itself a transcriptional target of p53, thus the activity of the guardian of the genome is regulated by a negative feedback mechanism [64].

When mild stress is perceived, MDM2 mono-ubiquitinates p53, triggering p53 de-stabilization and its nuclear export. Once activated, p53 induces downstream effector pathways, including cell cycle arrest, necessary for repairing mild DNA damage [65]. Under conditions where DNA repair cannot occur or if stress is too severe, the cell will undergo apoptosis. In this instance, p53 activates BCL2-associated X, apoptosis regulator (BAX), p53 upregulated modulator of apoptosis (PUMA; also known as BBC3), and NOXA (also known as PMAIP1), among others [66]. At the level of mitochondria, p53 can physically interact with multidomain anti-apoptotic (Bcl-xL and Bcl-2) and pro-apoptotic (Bak) Bcl-2 members inducing the permeabilization of the outer membrane of the mitochondrial membrane, a step necessary for the release of cytochrome c and induction of caspase-dependent apoptosis [61].

## 10. *TP*53 Mutations

The *TP*53 gene is often mutated in cancers, with somatic mutations occurring in more than half of all human cancers and the germline mutations associated with the Li–Fraumeni syndrome (LFS), a rare congenital condition that renders patients prone to the development of cancer at an early age [67].

Most *TP*53 mutations are of missense type (an exception among tumor suppressor genes) and are usually categorized as driver mutations in various cancer types. Often, *TP*53 mutations are linked to a worse prognosis and resistant disease. The majority of mutations occur within the DNA binding domain rendering the protein inactive or promoting the gain of new functions. DBD mutations are subdivided into DNA contact mutations and structural mutations [68]. Among the most common mutations there are: R175H, G245S, R248W, R249S, R273H, and R282W, which impede p53 activity by destabilizing and disrupting the p53 DBD [69,70]. Missense mutations can also result in the gain of functions effects [71]. The second most common types of mutation are nonsense mutations, indel mutations and other [72]. In cancer cases in which the *TP*53 gene is not mutated, the protein is subject to rapid cellular turnover, as it is ubiquitinated and degraded by the up-regulated or hyper-activated of MDM2 and MDMX proteins [61,65,73].

## 11. *TP*53- and *EGFR*-Mutated Lung Cancer

*TP*53 mutations and deletions are associated with treatment resistance in several cancer types, including acute lymphoblastic leukemia, melanoma [74], osteosarcoma [75], and breast cancer [76], as well as ovarian and testicular cancers [77,78]. Relevantly, *TP*53 is mutated in 8–47% of NSCLC patients who never smoked and in 26–71% in NSCLC smoking patients [79]. These patients face an increased rate of resistance to first-line chemotherapy, a more aggressive disease, and shortened survival rates [80,81].

Several studies have shown the association between mutations or deletion of the *TP*53 gene and EGFRi efficacy [82,83,84,85,86,87]: patients affected by concurrent *TP*53 and *EGFR* mutations exhibit lower responsiveness to EGFRi [83,84,87]. On the other hand, wild-type p53 increases tumor cells’ sensitivity to EGFRi, for instance, by boosting the Fas/FasL-mediated apoptotic signaling [86]. Different mutations in *TP*53 are associated with varying sensitivity and acquired resistance to EGFRi. As reported by Canale and collaborators, *TP*53 mutations, particularly those occurring within exon 8 (within DBD), were associated with a decreased sensitivity to EGFRi and a worse prognosis, especially in those individuals bearing *EGFR* exon 19 deletions [88].

The resistance to TKIs has been broadly studied, yet, due to recent approval, the resistance to frontline osimertinib is not fully understood, especially in the context of the clonal evolution. The most recent report in advanced NSCLC näive patients and in patients previously treated with 1st/2nd generation TKIs receiving osimertinib indicates that the most common EGFR-independent resistance mutation affects the *TP*53 gene [89]. The occurrence of clones harboring mutant *TP*53 with high variant allele frequency (VAF > 20%) in myelodysplastic syndrome patients and secondary acute myeloid leukemia patients treated with epigenetic therapy was linked to treatment resistance; yet, the clonal evolution pattern in those diseases is complex. What is more, a transient response to treatment was observed in *TP*53 mutant clones which is indicative of a persistent mutant p53 clone in the refractory/relapse disease [90].

Whether there is a similar pattern of mutant p53-driven clonal evolution in resistance to osimertinib still remains to be investigated, and no data has been made publicly available. Yet, the heterogeneity of lung tumors might require the thorough single cell multi-omics-based studies to address this phenomenon. As *TP*73 and *TP*63 are rarely mutated in cancers the analysis of the methylation status of P1 and P2 might contribute to our understanding of the resistance disease.

In addition to the above, some pre-clinical studies denoted that the p53 status reflects primary sensitivity and resistance to EGFRi in a cell-type-specific manner. In example, the authors analyzed three *EGFR*-mutated NSCLC cell lines, including PC-9 (p53-R248Q), HCC827 (p53-v218del), and H1975 (p53-R273H) [91]. Authors found that p53-R248Q did not influence the sensitivity and the acquired resistance to EGFRi in PC-9 cells, but the silencing of p53-v218del induced a primary resistance in HCC827 cells through AXL, a Tyro3-Axl-Mer (TAM) receptor tyrosine kinase that promotes growth, migration, aggregation, and anti-inflammation [92]. Instead, p53-R273H was found to be associated with EGFRi resistance through the induction of epithelial-to-mesenchymal-transition that correlated in patients to both a poor prognosis and acquired resistance to different types of chemotherapeutic agents [93,94,95,96,97,98,99,100].

In cancer cells mutant for *TP*53, mutant p53 protein promotes invasion and metastasis by enhancing integrin α5β1 and epidermal growth factor trafficking. The study by Muller and colleagues showed that mutant p53 inhibits EGFR lysosomal degradation and restores its membrane localization, therefore prolonging EGFR pathway activation. In addition, the same work demonstrates that cancer cell migration is driven by mutant p53 due to the inhibition of other p53 family proteins, TAp63 and TAp73 described in more detail below [101].

Apart from the transcription-dependent functions, p53 has also several cytoplasmic cellular activities including part-taking in regulation of autophagy, oxidative stress or metabolism. These extra-nuclear activities are dependent on post-translational modifications including mono-ubiquitination, phosphorylation or acetylation of p53. Cytoplasmic p53 translocates to mitochondria where it is de-ubiquitinated by HAUSP and induces the permeabilization of the outer membrane leading to apoptosis.

Another critical functions of cytoplasmic p53 are the regulation of autophagy, and vesicles and membrane trafficking. It has been shown that p53 is involved in clathrin-mediated receptor internalization [102]. Cytoplasmic wild-type p53 interacts with clathrin heavy chain and contrary to mutant p53, promotes enhanced lysosomal degradation of EGFR, thus leading to the inhibition of the EGFR signal transduction [103].

**Figure 2:**
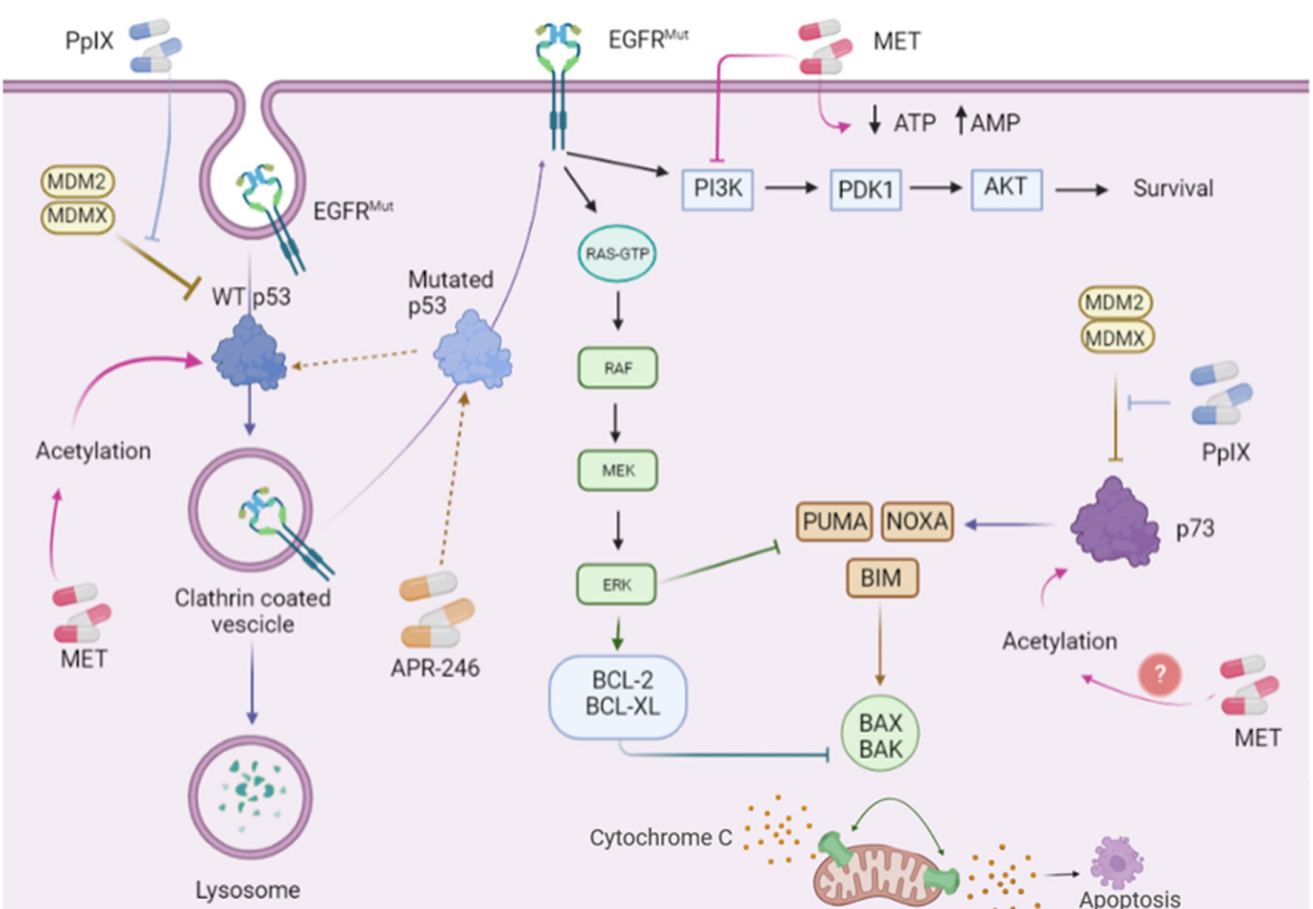
Reactivation of p53 protein family for improved therapy in *EGFR*-mutated (*EGFR*^mut^) lung cancer. Targeted drugs or repurposed drugs that reactivate p53 proteins in *EGFR*-mutated malignancies are promising candidates for improved cancer therapy. APR-246 (eprenetapopt) refolds mutant p53 to wild-type conformation and might induce lysosome-mediate degradation of mutant EGFR protein (EGFR^mut^). At the same time, repurposed protoporphyrin IX (PpIX) and metformin (MET) might promote reactivation of wild-type p53 and p73 by inhibiting their interactions with MDM2 and MDMX or activating acetylation of p53 and p73 respectively. The reactivation of both tumor suppressor proteins might enhance apoptosis induction in the presence of EGFR^mut^. In addition, it might be foreseen that metformin-mediated inhibition of EGFR-PI3K—AKT pathway will further enhance the response of EGFR-mutated cancer cells to the treatment.

Thus, a positive regulatory loop exists between mutant p53 and EGFR, which might be targeted therapeutically as described in detail below (Figure 2, and associated Table 1).

## 12. Pharmacological Reactivation of p53

The reinstatement of p53 in vivo induces effective tumor regression. APR-246, known under the commercial name as eprenetapopt^®^, is an innovative compound currently in Phase III clinical testing in the *TP*53-mutated myeloid malignancies (ClinicalTrials.gov Identifier: NCT03745716). APR-246 reactivates mutant p53 to wild-type conformation and induces p53-dependent cancer cells death. Upon spontaneous conversion into methyl-quinuclidinone (MQ), the drug acts as a Michael acceptor by targeting cysteine residues in the p53 core domain [105,115]. The binding induces the change of conformation of the p53 core domain to wild-type-like and triggers the pro-apoptotic activity of the refolded p53. APR-246 also has other cellular targets like thioredoxin reductase (TRXR), a component of the thioredoxin—thioredoxin reductase system, and it induces a potent accumulation of ROS by additional neutralization of glutathione [107,116,117]. Other compounds which reactivate mutant p53 include for example the FDA approved arsenic trioxide, which rescues p53 structural mutants [118] or the COTI-2 compound which binds to mutant p53, restores wild-type conformation and induces cancer cell apoptosis [119].

In cancers retaining the wild-type *TP*53 gene, the p53 protein can be rescued from rapid degradation and reactivated by targeting the p53/MDM2 interactions. Analogs of nutlin, MI compounds or AMG232, the rationally designed small molecule inhibitors that hamper MDM2 activity by outcompeting with p53, are currently being tested in a clinical setting with variable outcomes. In addition, nutlins are not effective against those tumors in which both *MDM*2 and *MDM*X (4) are over-expressed due to their low affinity for MDM4 [120,121,122]. An innovative strategy based on the dual inhibition of the p53/MDM2 and p53/MDMX interactions is now currently being developed. For this purpose, stapled peptides, such as ALRN-6924 [123] or allosteric modulators of p53 N-terminus as has recently been published can be utilized [124].

Apart from the rationally designed compounds, p53 can also be reactivated by repurposed drugs, medicines that have been approved for clinical use for other indications than cancer. For example, in cancer cells, metformin, an antidiabetic drug which has pleiotropic functions, inhibits mitochondrial complex I, shifts the levels of ATP, and increases the pool of AMP, leading to the activation of AMP-dependent kinase, (AMPK). The antineoplastic properties of metformin are thus attributed to the activation of AMPK and consequent downregulation of mammalian TOR complex 1 (mTORC1) and the IGF-1/AKT pathways and AMPK-mediated acetylation and reactivation of p53 [109,125] (Table 1).

Comprehensive studies have been performed with repurposed protoporphyrin IX (PpIX) and the analog, verteporfin (VP), both approved by the FDA for the treatment of non-oncological human diseases, revealing the reactivation of p53 and p73 in cancer cells without affecting normal cells [111,112]. PpIX is an allosteric activator of p53 [124] which binds to p53 and p73 N-terminus domains and inhibits p53/MDM2 and p53/MDMX interactions and p73/MDM2/MDMX complexes. Inhibition of interactions with MDM2 and MDM4 triggers the stabilization of p53 and p73 on the protein levels, reactivates their transcription function, and in consequence induces tumor cell apoptosis (Figure 2).

Since *TP*73 is rarely mutated in cancers, one of the latest promising strategies in targeting mutant p53 cancers, apart from reactivating mutant p53 itself, is represented by the reactivation of other p53 family members, including p73 [61]. This will be discussed in more detail below.

## 13. p53 Isoforms

p53, and other p53 protein family members, are expressed in isoforms. More recently, the altered expression of shorter isoforms of p53 has become more and more relevant as cancer biomarker or as a potential modifier of the full-length p53-mediated cellular responses to chemotherapeutics. Specifically, Δ133p53 isoforms, lacking the N-terminal domain, have been linked to different pro-oncogenic functions such as angiogenesis (particularly Δ133p53) [126], stemness (Δ133p53β) [127], proliferation (specifically Δ133p53α) [128] and invasion (Δ160p53 and Δ133p53β) [129,130]. Moreover, the elevated expression of Δ133p53 isoforms has been associated with cancer aggressiveness and worse prognosis both in the colon [131] as well as in prostate carcinomas [132]. Conversely, the impact of the other N-terminus truncated isoforms, e.g., Δ40p53 isoforms, is still debated, and conflicting evidence has been reported [133], while Δ160p53 isoforms are not well characterized yet, even if a role in cancer progression has been proposed [134]. The role of p53 isoforms in lung cancer has not been studied yet, and, thus, a better understanding of the role of the different p53 isoforms should be pursued, particularly in cancers maintaining a wild-type p53 but also in the case of mutant p53 cancers.

## 14. p73 Tumor Suppressor

p73 protein belongs to the p53 protein family, bears a high homology in the DNA binding domain to p53, and thus recognizes many of the p53 target genes involved in tumor suppression. Indeed, mice KO for TAp73 are viable yet tumor prone, and around 32% of the cohort develop lung adenocarcinomas [135].

The high structural and functional homology between p53 and p73 explains the TAp73-mediated cell cycle arrest and apoptosis through the transactivation of p53 target genes such as *PUMA*, *CDKN*1A, *NOXA*, or *BAX* upon stress stimuli including elevated ROS levels or DNA damage. Conversely, the ΔNp73, N-terminus truncated isoform, prevents apoptosis through the inhibition of the full-length p73 isoform or through the inhibition of p53-mediated transcription [136]. Therefore, it is recognized as oncogene. In addition to sharing classical p53 functions, p73 was also shown to play a role in regulating metabolism, senescence, and fertility [137].

Similar to p53, p73 protein activity is regulated by a wide range of post-translational modifications, such as ubiquitination, phosphorylation, acetylation, or sumoylation [138]. In addition, p73 transcriptional activity and stability, such as p53, are modulated by MDM2 and MDMX. Yet, the primary E3 ligase responsible for p73 cellular turnover is ITCH which requires MDM2 for E3 activity.

The role of p73 in lung cancer has not been broadly studied so far. Scarce reports indicated that several differentially methylated CpGs in the *TP*73 promoter have been identified. Daskalos and co-workers quantitatively pinned down the methylation levels of P1 and P2 *TP*73 promoters by pyrosequencing. In this work, P1 promoter was found to be rarely hyper-methylated (6.8%), the P2 promoter, instead, was found to be hypo-methylated (55.9%) in most NSCLC cases, especially in squamous cell carcinomas but also in adenocarcinomas. P1 hyper-methylation and P2 hypo-methylation were associated with the decreased TAp73 mRNA levels and increased ΔNp73 mRNA levels, respectively [139]. Relevantly, the treatment with the demethylating agent, azacytidine (AZA), was shown to restore the expression of p73 in the human lung squamous cell carcinoma cell line at the mRNA and the protein level. Elevated levels of p73 sensitized lung cancer cells to AZA, indicating the role of p73 in response to demethylating agents [140]. Notably, high levels of ∆Np73 isoforms were shown to be linked to poor prognosis and resistance toward first-line therapy in several cancer types [141,142]. In addition, the sensitivity to cisplatin was also reported to increase in vitro by the pre-treatment with a demethylating agent, decitabine which increased p73 on mRNA and protein levels [143].

Overall, p73 emerges as a relevant predictive and prognostic biomarker in cancer, yet, more detailed studies are needed to appreciate the role of p73 in lung cancer development, progression, and response to the treatment.

## 15. Pharmacological Reactivation of p73

The homology between p53 and p73 can be translated into the therapeutic setting, and analogous therapies can be exploited in order to reactivate p73 [61]. Indeed, if administered at higher doses, nutlin, first rationally-designed MDM2 inhibitor, was shown to reactivate TAp73 and induce cancer cells apoptosis [144]. Additionally, RNA-mediated silencing of ITCH E3 ligase was described as a promising approach to induce cell death through p73 protein stabilization and reactivation of p73-dependent cancer cells death [145]. Among other cellular mechanisms, p73 activity can be inhibited through direct interactions with mutant p53. A small molecule, RETRA, was discovered to target the mutp53/p73 complex and inhibit mutant p53 cancer cells’ growth both in vitro and in mouse xenografts [146]. The mechanism of tumor suppression was via activation of p73 and p73-driven apoptosis.

Importantly, repurposed drugs listed above, such as PpIX and PpIX analog, verteporfin, reactivate p73 in tumor cells and inhibit tumor growth in vivo [112]. To our knowledge, studies with repurposed drugs are the most advanced reports describing the feasibility of TAp73 reactivation for cancer therapy. Overall, p73 might be reactivated through similar means to p53 and serves as a feasible therapeutic target for improved cancer therapy.

## 16. p63 Tumor Suppressor

p63 is a transcription factor and another member of the p53 protein family. To some degree it resembles in structure and function p53, and can activate some of the p53 target genes after DNA damage exposure, yet has many p53-independent functions. Like p53 and p73, p63 is expressed in several isoforms, with two major ones being TA isoforms acting as tumor suppressors and ΔN isoforms acting as oncogenes, regulating stemness and epithelial cells differentiation. Detailed studies demonstrated that unlike p53 and p73, p63 is present in cells as an inactive, closed dimer thus, likely it requires another therapeutic approach for reactivation in cancer [147].

The role of p63 in cancer has remained obscure for several years, given its prominent role in development as documented by the fact that *Tp*63 knock-out which mice showed defects in most of the ectoderm-derived tissues, were lacking the epidermis, and died within a day from birth [148]. Next, the germline mutations in *TP*63 gene were associated with ectodermal dysplasia syndromes in humans [149]. However, studies with the TA-specific p63 knock-out mice harboring Ras-oncogene clearly demonstrated that TAp63 isoforms display tumor-suppressive functions through mediating p53-independent senescence [150]. In addition, TAp63 is also recognized as a metastasis suppressor through its transcriptional up-regulation of SHARP1, a protein able to degrade HIF1α oncogene via proteasomal pathway at an oxygen level- and VHL-independent manner [151]. These effects were shown to be blocked by the presence of mutant p53 and SMAD2/3, forming a ternary complex able to inhibit the functions of TAp63 as a tumor suppressor completely [152]. Importantly, studies from the Flores’ and Vousden’s groups demonstrated that TAp63 could suppress cancer metastasis via a coordinated up-regulation of Dicer and miR-130b and by blocking integrin recycling and EGFR, respectively [101,153].

Conversely, the ΔNp63 isoform has been proposed to work as an oncogene since it bypasses an oncogene-induced senescence to support cell growth [154], favors angiogenesis and tumor progression (both in neuroblastomas and osteosarcomas) [155], and it was found over-expressed in head and neck squamous cell carcinomas serving as a survival factor [156] and in other cancers. Furthermore, it has been demonstrated that *TP*53-deficient tumors require ΔNp63 to maintain metastatic potential [157]. In lung squamous cell carcinoma (LUSC), ΔNp63 is used as a diagnostic marker as it is significantly altered in around 44% of cases [158].

## 17. p63 in NSCLC

ΔNp63 is used for the diagnosis of LUSC [159] yet the role in LUAD has not been broadly tested. Recent work from Flores’ Lab shows that ΔNp63 is needed for the proliferation of LUAD progenitors and that it is required for the self-renewal and maintenance of these cells [160]. The authors also demonstrated that ΔNp63 is required for both tumor initiation and progression of NCLSC. The proposed mechanism involves the modulation of the epigenetic state via the ΔNp63-mediated regulation of a typical landscape of enhancer-associated genes (including BCL9L, KRT5, and ETV5) [160]. Despite the proven significance of ΔNp63 in lung cancer development and progression, currently, no therapeutic strategies have been proposed to target oncogenic ΔNp63 itself, and thus, this field remains largely unexplored.

## 18. Conclusions

Emerging clinical evidence supports the critical role of mutant p53 in the progression and resistance of *EGFR*-mutated lung cancer to TKIs. Since mutated p53 protein accumulates to a high degree in cancer cells, we speculate that mutant p53 might be an important therapeutic target in the sub-group of TKI-resistant NSCLC patients. Mechanistically, mutant p53 reactivated to wild-type protein by small molecules such as APR-246 will likely inhibit EGFR signaling through receptor degradation and induce apoptosis (Figure 2). The addition of repurposed drugs such as metformin or PpIX, or VP, will likely amplify the anti-cancer effect through further stabilization of refolded p53 by inhibiting p53/MDM2 interactions. We thus hypothesize that combination treatments targeting mutant p53 and EGFR pathway should be tested in the clinical setting. Apart from direct targeting of EGFR^mut^, e.g., with novel allosteric EGFR inhibitor, we foresee that the novel combination could be based on PI3K inhibition or inhibition of anti-apoptotic Bcl2 with venetoclax already approved for other indications.

Of note, one should take under the consideration that the dosage of the repurposed metformin, an anti-diabetic drug, must be adjusted for the oncological use. Systemic shift in the fasting plasma glucose levels after MET uptake may contribute to its antitumoral effect, yet, it is likely that it is the cellular effect of the drug that will drive the anti-tumoral function metformin.

*TP*73 is rarely mutated in cancers, can be reactivated via pharmacological means, and the p73 protein is therefore a promising therapeutic target in cancers harboring mutant p53. Yet, more advanced studies are needed to evaluate the clinical significance of restoration of the p53 family members for improved therapy in NSCLC patients who relapsed on TKIs.

## Figures and Tables

**Table 1:** Description of drug candidates for combination treatments of EGFR^mut^ NSCLC patients.

Drug	Mechanism of Action	FDA Approvals/Clinical Trials
APR-246 (eprenetapopt)	Binding to and refolding mutant p53 [104,105] Inhibition of thioredoxin reductase and glutaredoxin [106,107]	13 clinical trials registered in cancer [108]
Metformin (MET)	Inhibition of mitochondrial complex I) [109] Activation of AMP-dependent kinase [110]	400 clinical trials registered in cancer [108]
Protoporphyrin IX (PpIX)	Inhibition of p53/MDM2/MDM4 interactions [111] Inhibition of TAp73/MDM2/MDM4 interactions [112] Inhibition of thioredoxin reductase [113,114]	29 clinical trials registered in cancer [108]

## Data Availability

Not applicable.

## References

[1] Sung H., Ferlay J., Siegel R.L., Laversanne M., Soerjomataram I., Jemal A., Bray F. (2021). Global Cancer Statistics 2020: GLOBOCAN Estimates of Incidence and Mortality Worldwide for 36 Cancers in 185 Countries. CA Cancer J. Clin..

[2] Lung Cancer Now a Growing Public Health Threat|MDedge Hematology and Oncology. https://www.mdedge.com/hematology-oncology/article/252493/lung-cancer/lung-cancer-now-growing-public-health-threat.

[3] Siegel R.L., Miller K.D., Fuchs H.E., Jemal A. (2021). Cancer Statistics, 2021. CA Cancer J. Clin..

[4] Ryu W.-S. (2017). Adenoviruses. Molecular Virology of Human Pathogenic Viruses.

[5] (2021). Lung Cancer Fact Sheet. https://www.thoracic.org/about/global-public-health/firs/resources/world-lung-cancer-day-fact-sheet-2021.pdf.

[6] Wahbah M., Boroumand N., Castro C., El-Zeky F., Eltorky M. (2007). Changing trends in the distribution of the histologic types of lung cancer: A review of 4,439 cases. Ann. Diagn. Pathol..

[7] Chansky K., Detterbeck F.C., Nicholson A.G., Rusch V.W., Vallières E., Groome P., Kennedy C., Krasnik M., Peake M., Shemanski L. (2017). The IASLC Lung Cancer Staging Project: External Validation of the Revision of the TNM Stage Groupings in the Eighth Edition of the TNM Classification of Lung Cancer. J. Thorac. Oncol..

[8] Rami-Porta R., Bolejack V., Giroux D.J., Chansky K., Crowley J., Asamura H., Goldstraw P. (2014). The IASLC Lung Cancer Staging Project: The New Database to Inform the Eighth Edition of the TNM Classification of Lung Cancer. J. Thorac. Oncol..

[9] Nicholson A.G., Tsao M.S., Beasley M.B., Borczuk A.C., Brambilla E., Cooper W.A., Dacic S., Jain D., Kerr K.M., Lantuejoul S. (2021). The 2021 WHO Classification of Lung Tumors: Impact of Advances since 2015. J. Thorac. Oncol..

[10] Acs B., Rantalainen M., Hartman J. (2020). Artificial intelligence as the next step towards precision pathology. J. Intern. Med..

[11] Tunali I., Gillies R.J., Schabath M.B. (2021). Application of radiomics and artificial intelligence for lung cancer precision medicine. Cold Spring Harb. Perspect. Med..

[12] Rolfo C., Mack P.C., Scagliotti G.V., Baas P., Barlesi F., Bivona T.G., Herbst R.S., Mok T.S., Peled N., Pirker R. (2018). Liquid Biopsy for Advanced Non-Small Cell Lung Cancer (NSCLC): A Statement Paper from the IASLC. J. Thorac. Oncol..

[13] PDQ Adult Treatment Editorial Board (2002). Non-Small cell lung cancer treatment (PDQ^®^): Patient version. PDQ Cancer Information Summaries.

[14] Herbst R.S., Maddox A.-M., Rothenberg M.L., Small E.J., Rubin E.H., Baselga J., Rojo F., Hong W.K., Swaisland H., Averbuch S.D. (2002). Selective Oral Epidermal Growth Factor Receptor Tyrosine Kinase Inhibitor ZD1839 Is Generally Well-Tolerated and Has Activity in Non–Small-Cell Lung Cancer and Other Solid Tumors: Results of a Phase I Trial. J. Clin. Oncol..

[15] Herbst R.S., Prager D., Hermann R., Fehrenbacher L., Johnson B.E., Sandler A., Kris M., Tran H.T., Klein P., Li X. (2005). TRIBUTE: A Phase III Trial of Erlotinib Hydrochloride (OSI-774) Combined with Carboplatin and Paclitaxel Chemotherapy in Advanced Non–Small-Cell Lung Cancer. J. Clin. Oncol..

[16] Benesova L., Minarik M., Jancarikova D., Belsanova B., Pesek M. (2010). Multiplicity of EGFR and KRAS mutations in non-small cell lung cancer (NSCLC) patients treated with tyrosine kinase inhibitors. Anticancer Res..

[17] Shchedrenok V.V., Sovakov A.N., Sebelev K.I. (1986). Immediate and late results of treating compression forms of lumbar osteochondrosis by the technic of puncture fenestration and decompression of the intervertebral disks. Zhurnal Nevropatol. I Psikhiatrii Im. SS Korsakova.

[18] Frampton G.M., Ali S.M., Rosenzweig M., Chmielecki J., Lu X., Bauer T.M., Akimov M., Bufill J.A., Lee C., Jentz D. (2015). Activation of MET via Diverse Exon 14 Splicing Alterations Occurs in Multiple Tumor Types and Confers Clinical Sensitivity to MET Inhibitors. Cancer Discov..

[19] The Cancer Genome Atlas Research Network (2014). Comprehensive molecular profiling of lung adenocarcinoma. Nature.

[20] Zehir A., Benayed R., Shah R.H., Syed A., Middha S., Kim H.R., Srinivasan P., Gao J., Chakravarty D., Devlin S.M. (2017). Mutational landscape of metastatic cancer revealed from prospective clinical sequencing of 10,000 patients. Nat. Med..

[21] Jordan E.J., Kim H.R., Arcila M.E., Barron D., Chakravarty D., Gao J., Chang M.T., Ni A., Kundra R., Jonsson P. (2017). Prospective Comprehensive Molecular Characterization of Lung Adenocarcinomas for Efficient Patient Matching to Approved and Emerging Therapies. Cancer Discov..

[22] Ding L., Getz G., Wheeler D.A., Mardis E.R., McLellan M.D., Cibulskis K., Sougnez C., Greulich H., Muzny D.M., Morgan M.B. (2008). Somatic mutations affect key pathways in lung adenocarcinoma. Nature.

[23] Campbell J.D., Alexandrov A., Kim J., Wala J., Berger A.H., Pedamallu C.S., Shukla S.A., Guo G., Brooks A.N., Murray B.A. (2016). Distinct patterns of somatic genome alterations in lung adenocarcinomas and squamous cell carcinomas. Nat. Genet..

[24] Imielinski M., Berger A.H., Hammerman P.S., Hernandez B., Pugh T.J., Hodis E., Cho J., Suh J., Capelletti M., Sivachenko A. (2012). Mapping the Hallmarks of Lung Adenocarcinoma with Massively Parallel Sequencing. Cell.

[25] Mina M., Raynaud F., Tavernari D., Battistello E., Sungalee S., Saghafinia S., Laessle T., Sanchez-Vega F., Schultz N., Oricchio E. (2017). Conditional Selection of Genomic Alterations Dictates Cancer Evolution and Oncogenic Dependencies. Cancer Cell.

[26] Campbell P.J. (2017). Cliques and Schisms of Cancer Genes. Cancer Cell.

[27] Skoulidis F., Heymach J.V. (2019). Co-occurring genomic alterations in non-small-cell lung cancer biology and therapy. Nat. Rev. Cancer.

[28] Skoulidis F., Byers L.A., Diao L., Papadimitrakopoulou V.A., Tong P., Izzo J., Behrens C., Kadara H., Parra E.R., Canales J.R. (2015). Co-occurring Genomic Alterations Define Major Subsets of *KRAS*-Mutant Lung Adenocarcinoma with Distinct Biology, Immune Profiles, and Therapeutic Vulnerabilities. Cancer Discov..

[29] Helena A.Y., Suzawa K., Jordan E.J., Zehir A., Ni A., Kim H.R., Kris M.G., Hellmann M.D., Li B.T., Somwar R. (2018). Concurrent Alterations in EGFR-Mutant Lung Cancers Associated with Resistance to EGFR Kinase Inhibitors and Characterization of MTOR as a Mediator of Resistance. Clin. Cancer Res..

[30] Blakely C.M., Watkins T.B.K., Wu W., Gini B., Chabon J.J., McCoach C.E., McGranahan N., Wilson G.A., Birkbak N., Olivas V.R. (2017). Evolution and clinical impact of co-occurring genetic alterations in advanced-stage EGFR-mutant lung cancers. Nat. Genet..

[31] Sanchez-Vega F., Mina M., Armenia J., Chatila W.K., Luna A., La K.C., Dimitriadoy S., Liu D.L., Kantheti H.S., Saghafinia S. (2018). Oncogenic Signaling Pathways in The Cancer Genome Atlas. Cell.

[32] Ellrott K., Bailey M.H., Saksena G., Covington K.R., Kandoth C., Stewart C., Hess J., Ma S., Chiotti K.E., McLellan M. (2018). Scalable Open Science Approach for Mutation Calling of Tumor Exomes Using Multiple Genomic Pipelines. Cell Syst..

[33] Hoadley K.A., Yau C., Hinoue T., Wolf D.M., Lazar A.J., Drill E., Shen R., Taylor A.M., Cherniack A.D., Thorsson V. (2018). Cell-of-Origin Patterns Dominate the Molecular Classification of 10,000 Tumors from 33 Types of Cancer. Cell.

[34] Kadara H., Choi M., Zhang J., Parra E., Rodriguez-Canales J., Gaffney S., Zhao Z., Behrens C., Fujimoto J., Chow C. (2016). Whole-exome sequencing and immune profiling of early-stage lung adenocarcinoma with fully annotated clinical follow-up. Ann. Oncol..

[35] Voldborg B.R., Damstrup L., Spang-Thomsen M., Poulsen H.S. (1997). Epidermal growth factor receptor (EGFR) and EGFR mutations, function and possible role in clinical trials. Ann. Oncol..

[36] Gullick W.J., Marsden J.J., Whittle N., Ward B., Bobrow L., Waterfield M.D. (1986). Expression of epidermal growth factor receptors on human cervical, ovarian, and vulval carcinomas. Cancer Res..

[37] Roskoski R. (2014). The ErbB/HER family of protein-tyrosine kinases and cancer. Pharmacol. Res..

[38] Harrison P.T., Vyse S., Huang P.H. (2019). Rare epidermal growth factor receptor (EGFR) mutations in non-small cell lung cancer. Semin. Cancer Biol..

[39] Kobayashi Y., Mitsudomi T. (2016). Not all epidermal growth factor receptor mutations in lung cancer are created equal: Perspectives for individualized treatment strategy. Cancer Sci..

[40] Attili I., Passaro A., Pisapia P., Malapelle U., de Marinis F. (2022). Uncommon *EGFR* Compound Mutations in Non-Small Cell Lung Cancer (NSCLC): A Systematic Review of Available Evidence. Curr. Oncol..

[41] Kannan S., Pradhan M.R., Tiwari G., Tan W.-C., Chowbay B., Tan E.H., Tan D.S.-W., Verma C. (2017). Hydration effects on the efficacy of the Epidermal growth factor receptor kinase inhibitor afatinib. Sci. Rep..

[42] Yun C.-H., Boggon T.J., Li Y., Woo M.S., Greulich H., Meyerson M., Eck M.J. (2007). Structures of Lung Cancer-Derived EGFR Mutants and Inhibitor Complexes: Mechanism of Activation and Insights into Differential Inhibitor Sensitivity. Cancer Cell.

[43] Landau M., Ben-Tal N. (2008). Dynamic equilibrium between multiple active and inactive conformations explains regulation and oncogenic mutations in ErbB receptors. Biochim. Biophys. Acta.

[44] Eck M.J., Yun C.-H. (2010). Structural and mechanistic underpinnings of the differential drug sensitivity of EGFR mutations in non-small cell lung cancer. Biochim. Biophys. Acta.

[45] Carey K.D., Garton A.J., Romero M.S., Kahler J., Thomson S., Ross S., Park F., Haley J.D., Gibson N., Sliwkowski M.X. (2006). Kinetic Analysis of Epidermal Growth Factor Receptor Somatic Mutant Proteins Shows Increased Sensitivity to the Epidermal Growth Factor Receptor Tyrosine Kinase Inhibitor, Erlotinib. Cancer Res..

[46] Yu H.A., Arcila M.E., Rekhtman N., Sima C.S., Zakowski M.F., Pao W., Kris M.G., Miller V.A., Ladanyi M., Riely G.J. (2013). Analysis of Tumor Specimens at the Time of Acquired Resistance to EGFR-TKI Therapy in 155 Patients with EGFR-Mutant Lung Cancers. Clin. Cancer Res..

[47] Yun C.-H., Mengwasser K.E., Toms A.V., Woo M.S., Greulich H., Wong K.K., Meyerson M., Eck M.J. (2008). The T790M mutation in EGFR kinase causes drug resistance by increasing the affinity for ATP. Proc. Natl. Acad. Sci. USA.

[48] Sequist L.V., Besse B., Lynch T.J., Miller V.A., Wong K.K., Gitlitz B., Eaton K., Zacharchuk C., Freyman A., Powell C. (2010). Neratinib, an Irreversible Pan-ErbB Receptor Tyrosine Kinase Inhibitor: Results of a Phase II Trial in Patients with Advanced Non–Small-Cell Lung Cancer. J. Clin. Oncol..

[49] Miller V.A., Hirsh V., Cadranel J., Chen Y.-M., Park K., Kim S.-W., Zhou C., Su W.-C., Wang M., Sun Y. (2012). Afatinib versus placebo for patients with advanced, metastatic non-small-cell lung cancer after failure of erlotinib, gefitinib, or both, and one or two lines of chemotherapy (LUX-Lung 1): A phase 2b/3 randomised trial. Lancet Oncol..

[50] Katakami N., Atagi S., Goto K., Hida T., Horai T., Inoue A., Ichinose Y., Koboyashi K., Takeda K., Kiura K. (2013). LUX-Lung 4: A Phase II Trial of Afatinib in Patients with Advanced Non–Small-Cell Lung Cancer Who Progressed During Prior Treatment with Erlotinib, Gefitinib, or Both. J. Clin. Oncol..

[51] Schuler M., Yang J.C.-H., Park K., Kim J.-H., Bennouna J., Chen Y.-M., Chouaid C., De Marinis F., Feng J.-F., Grossi F. (2015). Afatinib beyond progression in patients with non-small-cell lung cancer following chemotherapy, erlotinib/gefitinib and afatinib: Phase III randomized LUX-Lung 5 trial. Ann. Oncol..

[52] Yosaatmadja Y., Silva S., Dickson J.M., Patterson A.V., Smaill J.B., Flanagan J.U., McKeage M.J., Squire C.J. (2015). Binding mode of the breakthrough inhibitor AZD9291 to epidermal growth factor receptor revealed. J. Struct. Biol..

[53] Soria J.-C., Ohe Y., Vansteenkiste J., Reungwetwattana T., Chewaskulyong B., Lee K.H., Dechaphunkul A., Imamura F., Nogami N., Kurata T. (2018). Osimertinib in Untreated EGFR-Mutated Advanced Non–Small-Cell Lung Cancer. N. Engl. J. Med..

[54] Tagrisso Significantly Improves Overall Survival in the Phase III FLAURA Trial for 1st-Line egfr-Mutated Non-Small Cell Lung Cancer. https://www.astrazeneca.com/media-centre/press-releases/2019/tagrisso-significantly-improves-overall-survival-in-the-phase-iii-flaura-trial-for-1st-line-egfr-mutated-non-small-cell-lung-cancer-09082019.html#!.

[55] Final FLAURA Results Demonstrate Overall Survival Benefit. https://www.esmo.org/oncology-news/final-flaura-results-demonstrate-overall-survival-benefit-with-osimertinib-over-tkis-in-advanced-nsclc.

[56] Leonetti A., Sharma S., Minari R., Perego P., Giovannetti E., Tiseo M. (2019). Resistance mechanisms to osimertinib in EGFR-mutated non-small cell lung cancer. Br. J. Cancer.

[57] Zawacka-Pankau J.E. (2022). The Role of p53 Family in Cancer. Cancers.

[58] Hafner A., Bulyk M.L., Jambhekar A., Lahav G. (2019). The multiple mechanisms that regulate p53 activity and cell fate. Nat. Rev. Mol. Cell Biol..

[59] Levine A.J. (2019). The many faces of p53: Something for everyone. J. Mol. Cell Biol..

[60] Janic A., Valente L.J., Wakefield M.J., Di Stefano L., Milla L., Wilcox S., Yang H., Tai L., Vandenberg C.J., Kueh A.J. (2018). DNA repair processes are critical mediators of p53-dependent tumor suppression. Nat. Med..

[61] Zawacka-Pankau J. (2020). The Undervalued Avenue to Reinstate Tumor Suppressor Functionality of the p53 Protein Family for Improved Cancer Therapy-Drug Repurposing. Cancers.

[62] Joseph T.W., Zaika A., Moll U.M. (2003). Nuclear and cytoplasmic degradation of endogenous p53 and HDM2 occurs during down-regulation of the p53 response after multiple types of DNA damage. FASEB J..

[63] Kruse J.-P., Gu W. (2009). Modes of p53 Regulation. Cell.

[64] Tournillon A.-S., López I., Malbert-Colas L., Findakly S., Naski N., Olivares-Illana V., Karakostis K., Vojtesek B., Nylander K., Fåhraeus R. (2016). p53 binds the mdmx mRNA and controls its translation. Oncogene.

[65] Levine A.J. (2020). p53: 800 million years of evolution and 40 years of discovery. Nat. Cancer.

[66] Vousden K.H., Prives C. (2009). Blinded by the Light: The Growing Complexity of p53. Cell.

[67] de Andrade K.C., Khincha P.P., Hatton J.N., Frone M.N., Wegman-Ostrosky T., Mai P.L., Best A.F., Savage S.A. (2021). Cancer incidence, patterns, and genotype–phenotype associations in individuals with pathogenic or likely pathogenic germline TP53 variants: An observational cohort study. Lancet Oncol..

[68] Bykov V.J.N., Eriksson S.E., Bianchi J., Wiman K.G. (2018). Targeting mutant p53 for efficient cancer therapy. Nat. Rev. Cancer.

[69] Kandoth C., McLellan M.D., Vandin F., Ye K., Niu B., Lu C., Xie M., Zhang Q., McMichael J.F., Wyczalkowski M.A. (2013). Mutational landscape and significance across 12 major cancer types. Nature.

[70] Martínez-Jiménez F., Muiños F., Sentís I., Deu-Pons J., Reyes-Salazar I., Arnedo-Pac C., Mularoni L., Pich O., Bonet J., Kranas H. (2020). A compendium of mutational cancer driver genes. Nat. Cancer.

[71] Stein Y., Rotter V., Aloni-Grinstein R. (2019). Gain-of-Function Mutant p53: All the Roads Lead to Tumorigenesis. Int. J. Mol. Sci..

[72] World Health Organization IARC TP53 Database. https://p53.fr.

[73] Jiang L., Zawacka-Pankau J. (2020). The p53/MDM2/MDMX-targeted therapies—A clinical synopsis. Cell Death Dis..

[74] Li G., Tang L., Zhou X., Tron V., Ho V. (1998). Chemotherapy-induced apoptosis in melanoma cells is p53 dependent. Melanoma Res..

[75] Asada N., Tsuchiya H., Tomita K. (2000). De novo deletions of p53 gene and wild-type p53 correlate with acquired cisplatin-resistance in human osteosarcoma OST cell line. Anticancer Res..

[76] Berns E.M., Foekens J.A., Vossen R., Look M.P., Devilee P., Henzen-Logmans S.C., Van Staveren I.L., Van Putten W.L., Inganäs M., Gelder M.E.M.-V. (2000). Complete sequencing of TP53 predicts poor response to systemic therapy of advanced breast cancer. Cancer Res..

[77] Houldsworth J., Xiao H., Murty V., Chen W., Ray B., Reuter V.E., Bosl G.J., Chaganti R. (1998). Human male germ cell tumor resistance to cisplatin is linked to TP53 gene mutation. Oncogene.

[78] Righetti S.C., Perego P., Corna E., Pierotti M.A., Zunino F. (1999). Emergence of p53 mutant cisplatin-resistant ovarian carcinoma cells following drug exposure: Spontaneously mutant selection. Cell Growth Differ..

[79] Mogi A., Kuwano H. (2011). TP53 mutations in nonsmall cell lung cancer. J. Biomed. Biotechnol..

[80] Custodio A.B., González-Larriba J.L., Bobokova J., Calles A., Álvarez R., Cuadrado E., Manzano A., Díaz-Rubio E. (2009). Prognostic and Predictive Markers of Benefit from Adjuvant Chemotherapy in Early-Stage Non-small Cell Lung Cancer. J. Thorac. Oncol..

[81] Tsao M.-S., Aviel-Ronen S., Ding K., Lau D., Liu N., Sakurada A., Whitehead M., Zhu C.-Q., Livingston R., Johnson D.H. (2007). Prognostic and Predictive Importance of p53 and RAS for Adjuvant Chemotherapy in Non–Small-Cell Lung Cancer. J. Clin. Oncol..

[82] Chang G.-C., Hsu S.-L., Tsai J.-R., Liang F.-P., Lin S.-Y., Sheu G.-T., Chen C.-Y. (2004). Molecular mechanisms of ZD1839-induced G1-cell cycle arrest and apoptosis in human lung adenocarcinoma A549 cells. Biochem. Pharmacol..

[83] Labbé C., Cabanero M., Korpanty G.J., Tomasini P., Doherty M.K., Mascaux C., Jao K., Pitcher B., Wang R., Pintilie M. (2017). Prognostic and predictive effects of TP53 co-mutation in patients with EGFR -mutated non-small cell lung cancer (NSCLC). Lung Cancer.

[84] Molina-Vila M.A., Bertran-Alamillo J., Gascó A., Mayo-De-Las-Casas C., Sánchez-Ronco M., Pujantell-Pastor L., Bonanno L., Favaretto A.G., Cardona A.F., Vergnenègre A. (2014). Nondisruptive p53 Mutations Are Associated with Shorter Survival in Patients with Advanced Non–Small Cell Lung Cancer. Clin. Cancer Res..

[85] Munsch D., Watanabe-Fukunaga R., Bourdon J.-C., Nagata S., May E., Yonish-Rouach E., Reisdorf P. (2000). Human and Mouse Fas (APO-1/CD95) Death Receptor Genes Each Contain a p53-responsive Element That Is Activated by p53 Mutants Unable to Induce Apoptosis. J. Biol. Chem..

[86] Rho J.K., Choi Y.J., Ryoo B.-Y., Na I.I., Yang S.H., Kim C.H., Lee J.C. (2007). p53 Enhances Gefitinib-Induced Growth Inhibition and Apoptosis by Regulation of Fas in Non–Small Cell Lung Cancer. Cancer Res..

[87] VanderLaan P., Rangachari D., Mockus S.M., Spotlow V., Reddi H.V., Malcolm J., Huberman M.S., Joseph L.J., Kobayashi S.S., Costa D.B. (2017). Mutations in TP53, PIK3CA, PTEN and other genes in EGFR mutated lung cancers: Correlation with clinical outcomes. Lung Cancer.

[88] Canale M., Petracci E., Delmonte A., Chiadini E., Dazzi C., Papi M., Capelli L., Casanova C., De Luigi N., Mariotti M. (2017). Impact of *TP53* Mutations on Outcome in *EGFR*-Mutated Patients Treated with First-Line Tyrosine Kinase Inhibitors. Clin. Cancer Res..

[89] Fuchs V., Roisman L., Kian W., Daniel L., Dudnik J., Nechushtan H., Goldstein I., Dvir A., Soussan-Gutman L., Grinberg R. (2021). The impact of osimertinib’ line on clonal evolution in EGFRm NSCLC through NGS-based liquid biopsy and overcoming strategies for resistance. Lung Cancer.

[90] Uy G.L., Duncavage E.J., Chang G.S., Jacoby M.A., Miller C.A., Shao J., Heath S., Elliott K., Reineck T., Fulton R.S. (2017). Dynamic changes in the clonal structure of MDS and AML in response to epigenetic therapy. Leukemia.

[91] Chou C.-W., Lin C.-H., Hsiao T.-H., Lo C.-C., Hsieh C.-Y., Huang C.-C., Sher Y.-P. (2019). Therapeutic effects of statins against lung adenocarcinoma via p53 mutant-mediated apoptosis. Sci. Rep..

[92] AXL Gene-GeneCards|UFO Protein|UFO Antibody. https://www.genecards.org/cgi-bin/carddisp.pl?gene=AXL.

[93] Bremnes R.M., Veve R., Gabrielson E., Hirsch F.R., Baron A., Bemis L., Gemmill R.M., Drabkin H.A., Franklin W.A. (2002). High-Throughput Tissue Microarray Analysis Used to Evaluate Biology and Prognostic Significance of the E-Cadherin Pathway in Non–Small-Cell Lung Cancer. J. Clin. Oncol..

[94] Deeb G., Wang J., Ramnath N., Slocum H.K., Wiseman S., Beck A., Tan D. (2004). Altered E-cadherin and epidermal growth factor receptor expressions are associated with patient survival in lung cancer: A study utilizing high-density tissue microarray and immunohistochemistry. Mod. Pathol..

[95] Rho J.K., Choi Y.J., Lee J.K., Ryoo B.-Y., Na I.I., Yang S.H., Kim C.H., Lee J.C. (2009). Epithelial to mesenchymal transition derived from repeated exposure to gefitinib determines the sensitivity to EGFR inhibitors in A549, a non-small cell lung cancer cell line. Lung Cancer.

[96] Ji W., Choi Y.J., Kang M.-H., Sung K.J., Kim D.H., Jung S., Choi C.-M., Lee J.C., Rho J.K. (2020). Efficacy of the CDK7 Inhibitor on EMT-Associated Resistance to 3rd Generation EGFR-TKIs in Non-Small Cell Lung Cancer Cell Lines. Cells.

[97] Fischer K.R., Durrans A., Lee S., Sheng J., Li F., Wong S.T.C., Choi H., El Rayes T., Ryu S., Troeger J. (2015). Epithelial-to-mesenchymal transition is not required for lung metastasis but contributes to chemoresistance. Nature.

[98] Holohan C., Van Schaeybroeck S., Longley D.B., Johnston P.G. (2013). Cancer drug resistance: An evolving paradigm. Nat. Rev. Cancer.

[99] Zheng X., Carstens J.L., Kim J., Scheible M., Kaye J., Sugimoto H., Wu C.-C., LeBleu V.S., Kalluri R. (2015). Epithelial-to-mesenchymal transition is dispensable for metastasis but induces chemoresistance in pancreatic cancer. Nature.

[100] Jung S., Kim D.H., Choi Y.J., Kim S.Y., Park H., Lee H., Choi C.-M., Sung Y.H., Lee J.C., Rho J.K. (2021). Contribution of p53 in sensitivity to EGFR tyrosine kinase inhibitors in non-small cell lung cancer. Sci. Rep..

[101] Muller P.A.J., Caswell P.T., Doyle B., Iwanicki M.P., Tan E.H., Karim S., Lukashchuk N., Gillespie D.A., Ludwig R.L., Gosselin P. (2009). Mutant p53 Drives Invasion by Promoting Integrin Recycling. Cell.

[102] Comel A., Sorrentino G., Capaci V., Del Sal G. (2014). The cytoplasmic side of p53’s oncosuppressive activities. FEBS Lett..

[103] Endo Y., Sugiyama A., Li S.-A., Ohmori K., Ohata H., Yoshida Y., Shibuya M., Takei K., Enari M., Taya Y. (2008). Regulation of clathrin-mediated endocytosis by p53. Genes Cells.

[104] Bykov V.J.N., Issaeva N., Shilov A., Hultcrantz M., Pugacheva E., Chumakov P., Bergman J., Wiman K.G., Selivanova G. (2002). Restoration of the tumor suppressor function to mutant p53 by a low-molecular-weight compound. Nat. Med..

[105] Lambert J.M., Gorzov P., Veprintsev D., Söderqvist M., Segerbäck D., Bergman J., Fersht A.R., Hainaut P., Wiman K.G., Bykov V.N. (2009). PRIMA-1 Reactivates Mutant p53 by Covalent Binding to the Core Domain. Cancer Cell.

[106] Haffo L., Lu J., Bykov V.J.N., Martin S.S., Ren X., Coppo L., Wiman K.G., Holmgren A. (2018). Inhibition of the glutaredoxin and thioredoxin systems and ribonucleotide reductase by mutant p53-targeting compound APR-246. Sci. Rep..

[107] Peng X., Zhang M.-Q., Conserva F., Hosny G., Selivanova G., Bykov V.J., Arnér E.S., Wiman K.G. (2017). APR-246/PRIMA-1MET inhibits thioredoxin reductase 1 and converts the enzyme to a dedicated NADPH oxidase. Cell Death Dis..

[108] clinicaltrials.gov.

[109] Owen M.R., Doran E., Halestrap A.P. (2000). Evidence that metformin exerts its anti-diabetic effects through inhibition of complex 1 of the mitochondrial respiratory chain. Biochem. J..

[110] Zakikhani M., Dowling R., Fantus I.G., Sonenberg N., Pollak M. (2006). Metformin is an AMP kinase-dependent growth inhibitor for breast cancer cells. Cancer Res..

[111] Jiang L., Malik N., Acedo P., Zawacka-Pankau J. (2019). Protoporphyrin IX is a dual inhibitor of p53/MDM2 and p53/MDM4 interactions and induces apoptosis in B-cell chronic lymphocytic leukemia cells. Cell Death Discov..

[112] Sznarkowska A., Kostecka A., Kawiak A., Acedo P., Lion M., Inga A., Zawacka-Pankau J. (2018). Reactivation of TAp73 tumor suppressor by protoporphyrin IX, a metabolite of aminolevulinic acid, induces apoptosis in TP53-deficient cancer cells. Cell Div..

[113] Prast-Nielsen S., Dexheimer T.S., Schultz L., Stafford W.C., Cheng Q., Xu J., Jadhav A., Arnér E.S.J., Simeonov A. (2011). Inhibition of thioredoxin reductase 1 by porphyrins and other small molecules identified by a high-throughput screening assay. Free Radic. Biol. Med..

[114] Acedo P., Fernandes A., Zawacka-Pankau J. (2019). Activation of TAp73 and inhibition of TrxR by Verteporfin for improved cancer therapy in TP53 mutant pancreatic tumors. Future Sci. OA.

[115] Zhang Q., Bykov V.J.N., Wiman K.G., Zawacka-Pankau J. (2018). APR-246 reactivates mutant p53 by targeting cysteines 124 and 277. Cell Death Dis..

[116] Mohell N., Alfredsson J., Fransson A., Uustalu M., Bystrom S., Gullbo J., Hallberg A., Bykov V.J.N., Bjorklund U., Wiman K. (2015). APR-246 overcomes resistance to cisplatin and doxorubicin in ovarian cancer cells. Cell Death Dis..

[117] Tessoulin B., Descamps G., Moreau P., Maïga S., Lodé L., Godon C., Lambot S.M., Oullier T., le Gouill S., Amiot M. (2014). PRIMA-1Met induces myeloma cell death independent of p53 by impairing the GSH/ROS balance. Blood.

[118] Chen S., Wu J.-L., Liang Y., Tang Y.-G., Song H.-X., Wu L.-L., Xing Y.-F., Yan N., Li Y.-T., Wang Z.-Y. (2020). Arsenic Trioxide Rescues Structural p53 Mutations through a Cryptic Allosteric Site. Cancer Cell.

[119] Synnott N.C., O’Connell D., Crown J., Duffy M.J. (2019). COTI-2 reactivates mutant p53 and inhibits growth of triple-negative breast cancer cells. Breast Cancer Res. Treat..

[120] Joseph T.L., Madhumalar A., Brown C.J., Lane D., Verma C.S. (2010). Differential binding of p53 and nutlin to MDM2 and MDMX: Computational studies. Cell Cycle.

[121] Marine J.-C., Francoz S., Maetens M., Wahl G.M., Toledo F., Lozano G. (2006). Keeping p53 in check: Essential and synergistic functions of Mdm2 and Mdm4. Cell Death Differ..

[122] Patton J.T., Mayo L.D., Singhi A.D., Gudkov A.V., Stark G.R., Jackson M.W. (2006). Levels of HdmX Expression Dictate the Sensitivity of Normal and Transformed Cells to Nutlin-3. Cancer Res..

[123] Carvajal L.A., Ben Neriah D., Senecal A., Benard L., Thiruthuvanathan V., Yatsenko T., Narayanagari S.-R., Wheat J.C., Todorova T.I., Mitchell K. (2018). Dual inhibition of MDMX and MDM2 as a therapeutic strategy in leukemia. Sci. Transl. Med..

[124] Grinkevich V.V., Vema A., Fawkner K., Issaeva N., Andreotti V., Dickinson E.R., Hedström E., Spinnler C., Inga A., Larsson L.-G. (2022). Novel Allosteric Mechanism of Dual p53/MDM2 and p53/MDM4 Inhibition by a Small Molecule. Front. Mol. Biosci..

[125] Pierotti M.A., Berrino F., Gariboldi M., Melani C., Mogavero A., Negri T., Pasanisi P., Pilotti S. (2012). Targeting metabolism for cancer treatment and prevention: Metformin, an old drug with multi-faceted effects. Oncogene.

[126] Bernard H., Garmy-Susini B., Ainaoui N., Van Den Berghe L., Peurichard A., Javerzat S., Bikfalvi A., Lane D.P., Bourdon J.-C., Prats A.-C. (2012). The p53 isoform, Δ133p53α, stimulates angiogenesis and tumour progression. Oncogene.

[127] Arsic N., Gadea G., Lagerqvist E.L., Busson M., Cahuzac N., Brock C., Hollande F., Gire V., Pannequin J., Roux P. (2015). The p53 Isoform Δ133p53β Promotes Cancer Stem Cell Potential. Stem Cell Rep..

[128] Fujita K., Mondal A.M., Horikawa I., Nguyen G.H., Kumamoto K., Sohn J.J., Bowman E.D., Mathe E.A., Schetter A.J., Pine S.R. (2009). p53 isoforms Δ133p53 and p53β are endogenous regulators of replicative cellular senescence. Nat. Cell Biol..

[129] Candeias M.M., Hagiwara M., Matsuda M. (2016). Cancer-specific mutations in p53 induce the translation of Δ160p53 promoting tumorigenesis. EMBO Rep..

[130] Gadea G., Arsic N., Fernandes K., Diot A., Joruiz S.M., Abdallah S., Meuray V., Vinot S., Anguille C., Remenyi J. (2016). TP53 drives invasion through expression of its Δ133p53β variant. eLife.

[131] Campbell H., Fleming N., Roth I., Mehta S., Wiles A., Williams G., Vennin C., Arsic N., Parkin A., Pajic M. (2018). ∆133p53 isoform promotes tumour invasion and metastasis via interleukin-6 activation of JAK-STAT and RhoA-ROCK signalling. Nat. Commun..

[132] Kazantseva M., Mehta S., Eiholzer R.A., Gimenez G., Bowie S., Campbell H., Reily-Bell A.L., Roth I., Ray S., Drummond C.J. (2019). The Δ133p53β isoform promotes an immunosuppressive environment leading to aggressive prostate cancer. Cell Death Dis..

[133] Steffens Reinhardt L., Zhang X., Wawruszak A., Groen K., De Iuliis G.N., Avery-Kiejda K.A. (2020). Good Cop, Bad Cop: Defining the Roles of Δ40p53 in Cancer and Aging. Cancers.

[134] Tadijan A., Precazzini F., Hanžić N., Radić M., Gavioli N., Vlašić I., Ozretić P., Pinto L., Škreblin L., Barban G. (2021). Altered Expression of Shorter p53 Family Isoforms Can Impact Melanoma Aggressiveness. Cancers.

[135] Tomasini R., Tsuchihara K., Wilhelm M., Fujitani M., Rufini A., Cheung C.C., Khan F., Itie-Youten A., Wakeham A., Tsao M.-S. (2008). TAp73 knockout shows genomic instability with infertility and tumor suppressor functions. Genes Dev..

[136] Pozniak C.D., Radinovic S., Yang A., McKeon F., Kaplan D.R., Miller F.D. (2000). An anti-apoptotic role for the p53 family member, p73, during developmental neuron death. Science.

[137] Agostini M., Annicchiarico-Petruzzelli M., Melino G., Rufini A. (2016). Metabolic pathways regulated by TAp73 in response to oxidative stress. Oncotarget.

[138] Conforti F., Sayan A.E., Sreekumar R., Sayan B.S. (2012). Regulation of p73 activity by post-translational modifications. Cell Death Dis..

[139] Daskalos A., Logotheti S., Markopoulou S., Xinarianos G., Gosney J.R., Kastania A.N., Zoumpourlis V., Field J.K., Liloglou T. (2011). Global DNA hypomethylation-induced ΔNp73 transcriptional activation in non-small cell lung cancer. Cancer Lett..

[140] Liu K., Zhuang X., Mai Z. (2012). p73 expression is associated with cellular chemosensitivity in human non-small cell lung cancer cell lines. Oncol. Lett..

[141] Domínguez G., García J.M., Peña C., Silva J., García V., Martínez L., Maximiano C., Gómez M.E., Rivera J.A., García-Andrade C. (2006). ΔTAp73 Upregulation Correlates with Poor Prognosis in Human Tumors: Putative In Vivo Network Involving p73 Isoforms, p53, and E2F-1. J. Clin. Oncol..

[142] Hofstetter G., Berger A., Chamson M., Müller-Holzner E., Reimer D., Ulmer H., Uramoto H., Marth C., Zeimet A.G., Zeillinger R. (2011). Clinical Relevance of TAp73 and ΔNp73 Protein Expression in Ovarian Cancer. Int. J. Gynecol. Pathol..

[143] Bunch B., Krishnan N., Greenspan R.D., Ramakrishnan S., Attwood K., Yan L., Qi Q., Wang D., Morrison C., Omilian A. (2019). TAp73 expression and P1 promoter methylation, a potential marker for chemoresponsiveness to cisplatin therapy and survival in muscle-invasive bladder cancer (MIBC). Cell Cycle.

[144] Lau L.M.S., Nugent J.K., Zhao X., Irwin M.S. (2008). HDM2 antagonist Nutlin-3 disrupts p73-HDM2 binding and enhances p73 function. Oncogene.

[145] Hansen T., Rossi M., Roperch J., Ansell K., Simpson K., Taylor D., Mathon N., Knight R., Melino G. (2007). Itch inhibition regulates chemosensitivity in vitro. Biochem. Biophys. Res. Commun..

[146] Kravchenko J.E., Ilyinskaya G.V., Komarov P.G., Agapova L.S., Kochetkov D.V., Strom E., Frolova E.I., Kovriga I., Gudkov A., Feinstein E. (2008). Small-molecule RETRA suppresses mutant p53-bearing cancer cells through a p73-dependent salvage pathway. Proc. Natl. Acad. Sci. USA.

[147] Luh L.M., Kehrloesser S., Deutsch G.B., Gebel J., Coutandin D., Schäfer B., Agostini M., Melino G., Dotsch V. (2013). Analysis of the oligomeric state and transactivation potential of TAp73α. Cell Death Differ..

[148] Yang A., Schweitzer R., Sun D., Kaghad M., Walker N., Bronson R.T., Tabin C., Sharpe A., Caput D., Crum C. (1999). p63 is essential for regenerative proliferation in limb, craniofacial and epithelial development. Nature.

[149] Celli J., Duijf P., Hamel B.C., Bamshad M., Kramer B., Smits A.P., Newbury-Ecob R., Hennekam R.C., Van Buggenhout G., van Haeringen A. (1999). Heterozygous Germline Mutations in the p53 Homolog p63 Are the Cause of EEC Syndrome. Cell.

[150] Guo X., Keyes W.M., Papazoglu C., Zuber J., Li W., Lowe S.W., Vogel H., Mills A.A. (2009). TAp63 induces senescence and suppresses tumorigenesis in vivo. Nat. Cell Biol..

[151] Montagner M., Enzo E., Forcato M., Zanconato F., Parenti A., Rampazzo E., Basso G., Leo G., Rosato A., Bicciato S. (2012). SHARP1 suppresses breast cancer metastasis by promoting degradation of hypoxia-inducible factors. Nature.

[152] Adorno M., Cordenonsi M., Montagner M., Dupont S., Wong C., Hann B., Solari A., Bobisse S., Rondina M.B., Guzzardo V. (2009). A Mutant-p53/Smad Complex Opposes p63 to Empower TGFβ-Induced Metastasis. Cell.

[153] Su X., Chakravarti D., Cho M.S., Liu L.-Z., Gi Y.J., Lin Y.-L., Leung M.L., El-Naggar A., Creighton C.J., Suraokar M.B. (2010). TAp63 suppresses metastasis through coordinate regulation of Dicer and miRNAs. Nature.

[154] Keyes W.M., Pecoraro M., Aranda V., Vernersson-Lindahl E., Li W., Vogel H., Guo X., Garcia E.L., Michurina T.V., Enikolopov G. (2011). ΔNp63α Is an Oncogene that Targets Chromatin Remodeler Lsh to Drive Skin Stem Cell Proliferation and Tumorigenesis. Cell Stem Cell.

[155] Bid H.K., Roberts R.D., Cam M., Audino A., Kurmasheva R.T., Lin J., Houghton P.J., Cam H. (2014). ΔNp63 Promotes Pediatric Neuroblastoma and Osteosarcoma by Regulating Tumor Angiogenesis. Cancer Res..

[156] Rocco J.W., Leong C.-O., Kuperwasser N., DeYoung M.P., Ellisen L.W. (2006). p63 mediates survival in squamous cell carcinoma by suppression of p73-dependent apoptosis. Cancer Cell.

[157] Venkatanarayan A., Raulji P., Norton W., Chakravarti D., Coarfa C., Su X., Sandur S.K., Ramirez M.S., Lee J., Kingsley C.V. (2014). IAPP-driven metabolic reprogramming induces regression of p53-deficient tumours in vivo. Nature.

[158] Conde E., Angulo B., Redondo P., Toldos O., García-García E., Suarez-Gauthier A., Rubio-Viqueira B., Marrón C., García-Luján R., Sánchez-Céspedes M. (2010). The Use of P63 Immunohistochemistry for the Identification of Squamous Cell Carcinoma of the Lung. PLoS ONE.

[159] Osmani L., Askin F., Gabrielson E., Li Q.K. (2018). Current WHO guidelines and the critical role of immunohistochemical markers in the subclassification of non-small cell lung carcinoma (NSCLC): Moving from targeted therapy to immunotherapy. Semin. Cancer Biol..

[160] Napoli M., Wu S.J., Gore B.L., Abbas H.A., Lee K., Checker R., Dhar S., Rajapakshe K., Tan A.C., Lee M.G. (2022). ΔNp63 regulates a common landscape of enhancer associated genes in non-small cell lung cancer. Nat. Commun..

